# Supplementary material for: Estimation of the future prevalence of diabetes based on data from the Brazilian Study of Cardiovascular Risk Factors in Adolescents (ERICA)
Source: PLoS One. 2025 Jun 24;20(6):e0326436. doi: 10.1371/journal.pone.0326436 (PMC12186920; doi:10.1371/journal.pone.0326436)
Supplement: S1 File — (PDF) [file pone.0326436.s001.pdf]

a. Medline

(("adult*"[Title/Abstract]) AND ((((("adolescent*" OR "adolescence") OR "youth*") OR "teenagers") OR "teenager") OR "children") OR "child"[Title/Abstract]) AND ((((((((((("factor*, risk" OR "risk factor*") OR "population* at risk") OR "risk scores") OR "risk score") OR "score, risk") OR "risk factor scores") OR "risk factor score") OR "score, risk factor"[Title/Abstract]) OR "health correlates") OR "correlates, health") AND ((((((((((((((((("diabetes mellitus, adult onset" OR "adult onset diabetes mellitus") OR "diabetes mellitus, ketosis resistant") OR "diabetes mellitus, non insulin dependent") OR "diabetes mellitus, noninsulin dependent") OR "non insulin dependent diabetes mellitus") OR "diabetes mellitus, stable") OR "diabetes mellitus, type ii") OR "niddm") OR "diabetes mellitus, noninsulin dependent") OR "diabetes mellitus, maturity onset") OR "maturity onset diabetes mellitus") OR "mody") OR "diabetes mellitus, type 2") OR "diabetes mellitus, slow onset") OR "slow onset diabetes mellitus") OR "diabetes, type 2"[Title/Abstract])))

(("adult") AND ("adolescent") AND ("risk factor") AND ("diabetes mellitus"))

b. Embase

('risk factor':ab,ti AND adolescent:ab,ti OR child:ab,ti) AND adult:ab,ti AND 'diabetes mellitus':ab,ti
