# Supplementary figures and images for: Estimation of the future prevalence of diabetes based on data from the Brazilian Study of Cardiovascular Risk Factors in Adolescents (ERICA)
Source: PLoS One. 2025 Jun 24;20(6):e0326436. doi: 10.1371/journal.pone.0326436 (PMC12186920; doi:10.1371/journal.pone.0326436)

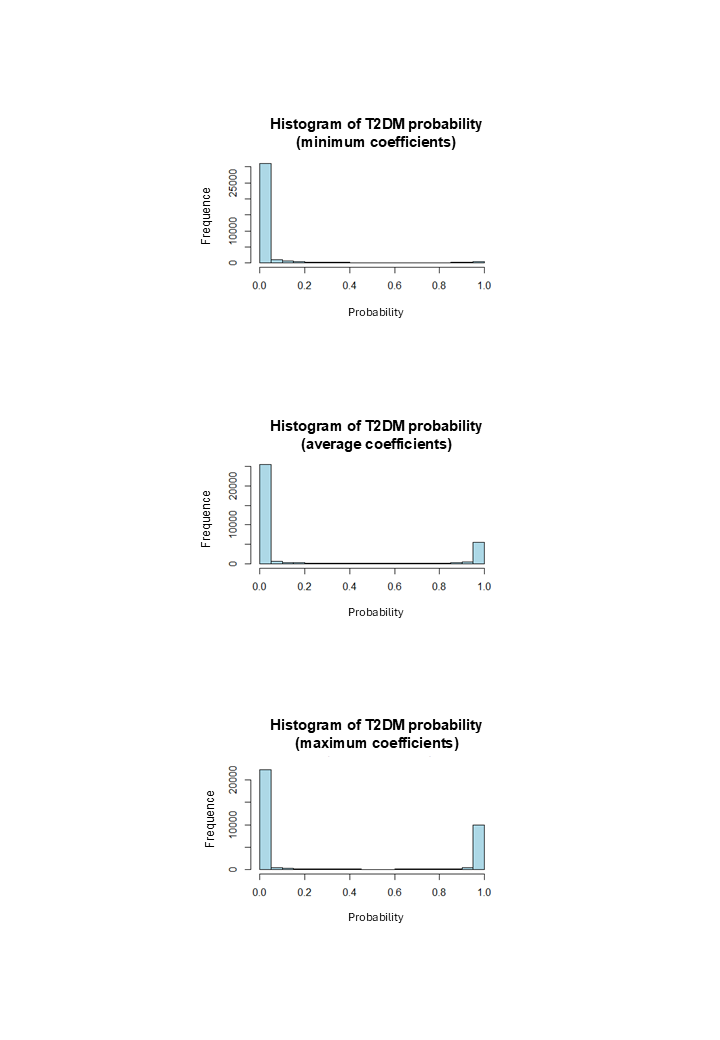

Supplement: S2 File — (TIF) [file pone.0326436.s002.tif]
