# Supplementary material for: Estimation of the future prevalence of diabetes based on data from the Brazilian Study of Cardiovascular Risk Factors in Adolescents (ERICA)
Source: PLoS One. 2025 Jun 24;20(6):e0326436. doi: 10.1371/journal.pone.0326436 (PMC12186920; doi:10.1371/journal.pone.0326436)
Supplement: S3 File — (PDF) [file pone.0326436.s003.pdf]

install.packages("haven") # --> Installs the package that imports the Stata database

install.packages("survey") # --> Installs the package that analyzes survey data

install.packages("readxl") # --> Installs the package that imports the morning post-stratification

install.packages("dplyr") # --> Installs the package that modifies the data

require("haven") # --> Loads the package that imports the Stata database

require("survey") # --> Loads the package that analyzes survey data

require("readxl") # --> Loads the package that imports the morning post-stratification

require("dplyr") # --> Loads the package that modifies the data

base <- read_dta('/media/marcelo/0C2A15692A1550D61/BKP/Documents/BKP/Dados/Bárbara (NATS)/Projeto_235/Projeto_Erica_235.dta', encoding = "latin1") # --> Imports the ERICA database

popmanha <- read_excel('/media/marcelo/0C2A15692A1550D61/BKP/Documents/BKP/Dados/Bárbara (NATS)/Projeto_235/Arquivo_complementar_R/Totalpopulacional_posestratificacao/Pop_manhã_com_sangue/popmanha_estrato_sexo_idade.xlsx') # --> Imports the post-stratification database

base_analise <- as.data.frame(base) # --> Converts the imported database to the ERICA analysis dataset

popmanha_analise <- as.data.frame(popmanha) # --> Converts the imported database to the post-stratification analysis dataset

base_analise$concat <- paste(base_analise$estrato_geo, base_analise$sexo, base_analise$idade_est, sep = "") # --> Creates the column that concatenates stratum, gender, and age

base_analise <- left_join(base_analise, popmanha_analise, by = "concat") # --> Joins the post-stratification column to the analysis dataset

base_analise <- subset(base_analise, diabete == 2) # --> Subsets the data, excluding individuals with diabetes

```
desenho <- svydesign(data=base_analise, ids=~cod_UPA, strata=~cod_estr_sel,
nest=TRUE, weights=~pesonat) # --> Creates the pre-stratification design
```

```
pop.types <- data.frame(posest=base_analise$estratop,
Freq=as.numeric(base_analise$pop_manha)) # --> Creates the population weight for
post-stratification

posest <- base_analise$estratopos # --> Extracts the post-stratum for the post-
stratification design

desenho.post <- postStratify(design=desenho, strata=~posest, population=pop.types) #
--> Creates the post-stratification design
```

```
medias <- svyby(~idade_est.x + ativfisminsem + pesokg + imcfim + medcc + sist +
diast + colest_total + colest_hdl + colest_ldl + triglic + glicose + hb_glicos + insulina +
homair, ~ sexo.x, desenho.post, svymean, keep.var=TRUE, na.rm=T) #--> Calculates
the means using the post-stratified weights
```

```
medias #--> Displays the results of the 'medias' object
```

```
a <- svymean(~interaction(sexo.x, regbr), design = desenho.post) # --> Calculates the
proportions of the selected variable responses by gender

b <- ftable(a, rownames = list(sexo.x = c("F", "M"), regbr =
levels(as.factor(base_analise$regbr)))) # --> Converts the 'a' object into a summary
table by gender

round(100 * b, 2) # --> Rounds the table to two decimal places
```

```
a <- svymean(~interaction(sexo.x, esc_tipo), design = desenho.post) # --> Calculates
the proportions of the selected variable responses by gender

b <- ftable(a, rownames = list(sexo.x = c("F", "M"), esc_tipo =
levels(as.factor(base_analise$esc_tipo)))) # --> Converts the 'a' object into a summary
table by gender

round(100 * b, 2) # --> Rounds the table to two decimal places
```

```
# List of other variables:

"regbr" "esc_tipo" "cor"  "fuma" "quantoscigarros"  "hipertensao"  "estadonut"
"result_pa" "ha" "catcolest" "cathdl" "catldl" "cattrig" "catins" "catglic" "cathbg"
"classeeco" "ccalt" "trigalt" "hdlalt" "glicalt" "paalt" "sindmet"

"hapai" "hamae" "diabpai" "diabmae"
```
