# Supplementary material for: Estimation of the future prevalence of diabetes based on data from the Brazilian Study of Cardiovascular Risk Factors in Adolescents (ERICA)
Source: PLoS One. 2025 Jun 24;20(6):e0326436. doi: 10.1371/journal.pone.0326436 (PMC12186920; doi:10.1371/journal.pone.0326436)
Supplement: S4 File — (PDF) [file pone.0326436.s004.pdf]

```
library(readxl)
library(dplyr)
library(ggplot2)
library(purrr)
library(data.table)
library(stringr)

# List of coefficients
inputs <- list (
  intercept = list(coefficient = 0.0918, sd_c = 0, average = NA),
  bmi = list(coefficient = 1.55, sd_c = 0.23, average = 16.95),
  hdl_c = list(coefficient = 1.37, sd_c = 0.19, average = 66.14),
  spb = list(coefficient = 0.94, sd_c = 0.16, average = 98.46),
  dbp = list(coefficient = 1.15, sd_c = 0.18, average = 50.95),
  tc = list(coefficient = 1.16, sd_c = 0.17, average = 164.08),
  tg = list(coefficient = 1, sd_c = 0.16, average = 66.76),
  ldl_c = list(coefficient = 1.3, sd_c = 0.18, average = 90.43)
)

# Import db_final_semDM dataset

# Probabilistic sensitivity analysis

# Create the coefficient table, add variable names, and create max and min columns
coefficient_table <- rbindlist(inputs)
coefficient_table <- coefficient_table %>%
  mutate(coef_name = names(inputs))

adjusted_coef_table <- coefficient_table %>%
  mutate(param_max = log(coefficient + sd_c),
         param_min = log(coefficient - sd_c))

# Create vectors for sensitivity analysis with minimum and maximum coefficient values
(simultaneous variation)
min_vector <- adjusted_coef_table$param_min
max_vector <- adjusted_coef_table$param_max
avg_vector <- log(adjusted_coef_table$coefficient)

# Create a matrix with variables to multiply by the vectors
variable_matrix <- db_final_semDM %>%
  mutate(intercept = 1,
         delta_bmi = imcfim - 16.95,
         delta_hdl = colest_hdl - 66.14,
         delta_sbp = sist - 98.46,
         delta_dbp = diast - 50.95,
         delta_tc = colest_total - 164.08,
         delta_tg = triglic - 66.76,
         delta_ldl = colest_ldl - 90.43) %>%
  select(intercept, delta_bmi, delta_hdl, delta_sbp, delta_dbp,
         delta_tc, delta_tg, delta_ldl) %>%
  as.matrix()
```

```
# Perform vector * matrix multiplication operations
predicted_values <- db_final_semDM %>%
  mutate(predicted_value_min = (variable_matrix %*% (min_vector))[, 1],
        predicted_value_max = (variable_matrix %*% (max_vector))[, 1],
        predicted_value_avg = (variable_matrix %*% (avg_vector))[, 1]) %>%
  mutate(exp_min = exp(predicted_value_min),
        exp_max = exp(predicted_value_max),
        exp_avg = exp(predicted_value_avg)) %>%
  mutate(prob_min = exp_min / (1 + exp_min),
        prob_max = exp_max / (1 + exp_max),
        prob_avg = exp_avg / (1 + exp_avg)) %>%
  mutate(future_dm_min = if_else(prob_min > 0.95, 1, 0),
        future_dm_max = if_else(prob_max > 0.95, 1, 0),
        future_dm_avg = if_else(prob_avg > 0.95, 1, 0))

final_table <- predicted_values %>%
  summarise(individuals_min = sum(predicted_values$future_dm_min, na.rm = TRUE),
          individuals_max = sum(predicted_values$future_dm_max, na.rm = TRUE),
          individuals_avg = sum(predicted_values$future_dm_avg, na.rm = TRUE))

# Deterministic sensitivity analysis

deterministic_analysis <- function(coef_name) {

  adjusted_coef_table <- coefficient_table %>%
    mutate(param_max = if_else(coef_name == coef_name, log(coefficient + sd_c),
log(coefficient)),
          param_min = if_else(coef_name == coef_name, log(coefficient - sd_c),
log(coefficient)))

  # Create vectors for sensitivity analysis with minimum and maximum coefficient
values (simultaneous variation)
  min_vector <- adjusted_coef_table$param_min
  max_vector <- adjusted_coef_table$param_max
  avg_vector <- log(adjusted_coef_table$coefficient)

  # Perform vector * matrix multiplication operations
  predicted_values <- db_final_semDM %>%
    mutate(predicted_value_min = (variable_matrix %*% (min_vector))[, 1],
          predicted_value_max = (variable_matrix %*% (max_vector))[, 1],
          predicted_value_avg = (variable_matrix %*% (avg_vector))[, 1]) %>%
    mutate(exp_min = exp(predicted_value_min),
          exp_max = exp(predicted_value_max),
          exp_avg = exp(predicted_value_avg)) %>%
    mutate(prob_min = exp_min / (1 + exp_min),
          prob_max = exp_max / (1 + exp_max),
          prob_avg = exp_avg / (1 + exp_avg)) %>%
    mutate(future_dm_min = if_else(prob_min > 0.95, 1, 0),
          future_dm_max = if_else(prob_max > 0.95, 1, 0),
          future_dm_avg = if_else(prob_avg > 0.95, 1, 0)) %>%
```

```
    summarise(individuals_min = sum(future_dm_min, na.rm = TRUE),
              individuals_max = sum(future_dm_max, na.rm = TRUE),
              individuals_avg = sum(future_dm_avg, na.rm = TRUE)) %>%
    mutate(coef_name = coef_name)

  return(predicted_values)

}

coef_names <- names(inputs) %>%
  str_subset("intercept", negate = TRUE)

deterministic_tables <- map(coef_names, deterministic_analysis)

final_deterministic_table <- rbindlist(deterministic_tables)
```
