# Supplementary material for: Estimation of the future prevalence of diabetes based on data from the Brazilian Study of Cardiovascular Risk Factors in Adolescents (ERICA)
Source: PLoS One. 2025 Jun 24;20(6):e0326436. doi: 10.1371/journal.pone.0326436 (PMC12186920; doi:10.1371/journal.pone.0326436)
Supplement: S5 File — (DOCX) [file pone.0326436.s005.docx]

| Study | Reasons for exclusion |
| --- | --- |
| Hippisley-Cox, Julia, and Carol Coupland. Development and validation of QDiabetes-2018 risk prediction algorithm to estimate future risk of type 2 diabetes: cohort study. *BMJ* 359 (2017). | - Study focuses on complications of T2DM rather than predicting its development from adolescent risk factors. - Population includes adults with established T2DM, not adolescents. - Model validation is based on microvascular and cardiovascular complications, rather than T2DM incidence. |
| Kazemian, Pooyan, et al. Development and validation of PREDICT-DM: a new microsimulation model to project and evaluate complications and treatments of type 2 diabetes mellitus. *Diabetes Technology & Therapeutics* 21.6 (2019): 344-355. | - Study is based on a microsimulation model for disease progression and treatment impact, rather than predictive modeling for new cases of T2DM. - Uses data from clinical trials with adult patients already diagnosed with diabetes, not adolescents. - Focuses on the validation of the simulation model rather than identifying early-life risk factors. |
| Basu, Sanjay, et al. Development and validation of Risk Equations for Complications Of type 2 Diabetes (RECODe) using individual participant data from randomised trials. *The lancet Diabetes & endocrinology* 5.10 (2017): 788-798. | - Developed using a general adult population (ages 25-84), not adolescents. - Model incorporates clinical data such as fasting glucose and HbA1c, which were not consistently available in ERICA. - Primarily aims to refine risk prediction in primary care settings, not in adolescent cohorts. |
| Sinaiko, Alan R., et al. The International Childhood Cardiovascular Cohort (i3C) consortium outcomes study of childhood cardiovascular risk factors and adult cardiovascular morbidity and mortality: Design and recruitment. *Contemporary clinical trials* 69 (2018): 55-64. | - Focuses on blood pressure and metabolic syndrome rather than direct prediction of T2DM. - Does not develop a predictive model for T2DM incidence based on adolescent cardiovascular risk factors. - Study population characteristics do not align with the ERICA dataset requirements. |
