# Supplementary material for: Estimation of the future prevalence of diabetes based on data from the Brazilian Study of Cardiovascular Risk Factors in Adolescents (ERICA)
Source: PLoS One. 2025 Jun 24;20(6):e0326436. doi: 10.1371/journal.pone.0326436 (PMC12186920; doi:10.1371/journal.pone.0326436)
Supplement: Attachment — Submitted filename: Response to reviewers.docx [file pone.0326436.s007.docx]

Dear Editor and Reviewers,

We have carefully reviewed the formatting of our manuscript to ensure compliance with PLOS ONE's style guidelines, and it appears to be in accordance with the provided templates.

Regarding data availability, our study utilized third-party data sources, including the ERICA Study, Vigitel, and the Bogalusa Heart Study. As we do not own these datasets, we are unable to publicly share the raw data. However, we have updated the manuscript to clarify the process for accessing these datasets within the Data Availability Statement section, where we provide the necessary information for researchers to request access through the respective data providers. Additionally, Figure 2 was created entirely by the authors. To ensure compliance with CC BY 4.0 licensing, we have included the following statement in its figure legend: "This figure was created by the authors and is published under the Creative Commons Attribution 4.0 International (CC BY 4.0) license." These revisions ensure that our manuscript meets PLOS ONE’s formatting, data accessibility, and copyright requirements.

Moreover, we sincerely appreciate the time and effort the reviewers and editorial team have dedicated to evaluating our manuscript. Their constructive feedback has been invaluable in improving the clarity, methodological rigor, and overall quality of our study. Below, we provide a point-by-point response to the comments raised by Reviewers 2 and 3. We have carefully addressed each suggestion and made the necessary modifications to the manuscript.

For clarity, the reviewers' comments are presented in bold, followed by our responses in regular text. Additionally, all referenced line numbers correspond to the revised manuscript with tracked changes, ensuring transparency in the modifications made.

**Reviewer #2** 
**1. In the abstraction section, please explain more the objective of this study.**

We revised the abstract and the introduction to better describe the study's objective, methodology, and significance. The new version explicitly states that the study applies a predictive model to ERICA’s sample to estimate the likelihood of developing type 2 diabetes mellitus (T2DM) in adulthood.

The abstract was slightly modified, so that the sentence on the study’s goal was changed from lines 34 to 36: *“This paper aims to estimate the future prevalence type 2 diabetes mellitus on individuals from the Study of Cardiovascular Risk Factors in Adolescents (ERICA) sample, based on the progression of their cardiovascular risk factors”*

The revised introduction now reads on lines 127-129: “*Given the increasing impact of T2DM on public health, this study aims to estimate its future prevalence on ERICA’s representative sample of adolescents by projecting the progression of their baseline CVRF and the resulting likelihood of developing T2DM.*”

**2. Line 53 and 54, please add references for this sentence.**

We have included the following reference to support this statement: *The Expert Committee on the Diagnosis and Classification of Diabetes Mellitus. Report of the Expert Committee on the Diagnosis and Classification of Diabetes Mellitus. Diabetes Care [Internet]. 1997 Jul [cited 2021 Oct 3];20(7):1183–97. Available from: https://care.diabetesjournals.org/content/20/7/1183.*

**3. "Diabetes-related costs in Brazil were calculated at BRL 52.3 billion in 2019…" before this sentence you can mention in one sentence that " Various factors such as genetics, nutrition factors, inactivity and other environmental factors are involved in diabetes etiology."you can read and add these articles as references to this sentence: "Association of circulating adipokines with metabolic dyslipidemia in obese versus non-obese individuals" and " Effect of Calcium and Vitamin D Co-supplementation on Blood Pressure: A Systematic Review and Meta-Analysis"**

We have carefully evaluated the two references suggested by Reviewer 2 and, after detailed consideration, we have decided not to include them in our revised manuscript for the reasons outlined below.

The first suggested reference, by Morvaridzadeh et al. (2020), is a systematic review and meta-analysis evaluating the effects of calcium and vitamin D co-supplementation on blood pressure regulation. While the study finds a modest reduction in diastolic blood pressure, it does not establish a direct connection between these nutrients and type 2 diabetes mellitus (T2DM) risk. Our study focuses on estimating the future prevalence of T2DM using cardiovascular risk factors in adolescents, specifically analyzing variables such as triglycerides, LDL cholesterol, and blood pressure. Although hypertension is a known risk factor for diabetes, our research does not examine dietary supplementation as an intervention, nor does it explore the specific role of vitamin D and calcium in diabetes prediction. Given that the focus of our study is on predictive modeling rather than nutritional interventions for blood pressure control, citing this reference would not contribute to the broader epidemiological and policy-related context that we establish to justify our study.

The second reference, by Rahimlou et al. (2015), investigates the association between circulating adipokines, including retinol-binding protein 4 (RBP4), vaspin, and omentin-1, and metabolic dyslipidemia in obese and non-obese individuals. The study concludes that higher RBP4 levels are associated with an increased risk of metabolic dyslipidemia, which is itself a known risk factor for cardiovascular diseases and diabetes development. While our predictive model includes certain markers of dyslipidemia, such as triglycerides and LDL cholesterol, it does not incorporate RBP4, vaspin, or omentin-1 as variables. Our introduction is structured to provide a broad epidemiological background on T2DM, highlighting its increasing prevalence, economic burden, and relevance for public policy. It does not delve into specific biomarkers or mechanisms related to metabolic dyslipidemia, as this level of detail is more relevant in the methodological and discussion sections. Therefore, citing this reference in the introduction would not be appropriate within the current structure of our introduction.

Considering the objectives and focus of this section of the manuscript, we believe that these references do not directly contribute to the contextual framework we establish for our study. We appreciate the reviewers’ suggestions and remain open to further discussion if additional clarification is needed.

**4. In the introduction section and paragraph 3, the transition from the global impact of diabetes to the objectives of the study is somewhat abrupt. It would benefit from a clearer lead-in statement, outlining the purpose of this particular research in the context of existing studies.**

Reviewing the introduction section and paragraph 3, we believe the concern was related to the need for a stronger connection between existing projections and the justification for using ERICA data. To improve clarity, we have refined the transition to explicitly highlight the gap in current estimates — namely, that they do not integrate adolescent cardiovascular risk factors into predictive models. The revised text now states in lines 94-101: “*Such a trend for increased prevalence, and financial impact, of T2DM is an important element for planning public policies. Literature provides varied projections, as the aforementioned data from the IDF and the IHME. The Ministry of Health also projects, based on Vigitel data, a growth in the prevalence of diabetes of about 9% (2.8% - 15.4%) for 2032 [4]. These data provide broad estimates based on a general population trend but do not specifically account for adolescent cardiovascular risk factors. Analyzing the T2DM tendency in a representative adolescent sample and considering their specific risk factors into the models could provide more precise estimates for the future generation.”* This revision ensures that the transition not only links global/national projections to the study's focus but also clearly identifies the research gap our study addresses.

**5. it is not clear why specific predictive models from the literature were chosen over others. Expanding on the justification for the model selection could strengthen the rigor of the method.**

We believe the manuscript already provides a clear justification for the selection of the predictive model. In the Methods section, the selection criteria are explicitly outlined, but we made a few changes to improve clarity:

- The sentence on lines 137-140: *“Evidence was sought focusing on studies that correlated childhood or adolescent CVRF with the development of T2DM in adulthood. Eligible studies were those that analyzed CVRF measured during childhood or adolescence, and also available for ERICA’s sample, with the aim of predicting the development of T2DM in adulthood.”* clarifies that only studies with a predictive focus were considered.
- The exclusion criteria are detailed in lines 142-152, right after the adjusted sentence *“Abstracts and articles selected for full reading were excluded if”,* followed by specific reasons such as incompatibility with ERICA’s dataset, reliance on unavailable variables, single-factor analysis, or non-T2DM outcomes.

In the Results section, the selection process and exclusion of other studies are explicitly addressed:

- The sentence in lines 276-277: *“The literature search resulted in 2,024 articles, of which only one was eligible for inclusion.”* highlights the rigorous selection process.
- The rationale for excluding other studies is reinforced in lines 281-283: *“The selected paper, 'Variabilities in Childhood Cardiovascular Risk Factors and Incident Diabetes in Adulthood: The Bogalusa Heart Study', utilized data on CVRF to predict T2DM in adulthood.”*, indicating that this was the only study that met all predefined criteria.

To further enhance transparency, a supplementary material file (*S5 File. Studies excluded after full-text review and reasons for non-eligibility*) will be included listing the four studies reviewed in full text before exclusion, providing additional details on the selection process.

**6. What was the inclusion criteria to choose the studies?**

We have carefully reviewed the section in question and identified a potential ambiguity in the wording that may have led to confusion. In our original manuscript, the sentence *"Eligible studies were those that used data from children or adolescents as predictive factors for T2DM."* may have been interpreted too broadly, suggesting that any study using pediatric or adolescent data was considered eligible, rather than specifying that the study had to establish a predictive relationship between childhood/adolescent cardiovascular risk factors (CVRF) and T2DM development in adulthood. To clarify this, we have revised the sentence to explicitly state that eligible studies were required to have a predictive focus, ensuring a clear correlation between early-life cardiovascular risk factors measurements and future disease outcomes. The revised sentence now reads on lines 138-140: *“Eligible studies were those that analyzed CVRF measured during childhood or adolescence, and also available for ERICA’s sample, with the aim of predicting the development of T2DM in adulthood.”* This correction aligns with the original intent of our study selection process, which sought models that could be applied to our dataset from the ERICA study. The revision removes any potential ambiguity and ensures that the criteria for model selection are clear and directly linked to the research objective.

**7. In term of logistic regression, please more explain the selected variables**

To address this point, we have explicitly included in:

- Lines 156-157 the following sentence: *“As per the inclusion criteria, the model incorporates risk factors that are available on ERICA”.*
- Lines 247-250 the following sentences: “*The selected model was designed based on the evolution of individuals’ CVRF – BMI, total cholesterol, HDL cholesterol, HDL cholesterol, triglycerides, SBP, and DBP – to explain the development of T2DM. The same CVRF are available for ERICA’s individuals, which enabled the model to be applied to them.”*  This addition ensures that the rationale for variable selection is clearly stated, making it explicit that the model incorporates well-established cardiovascular risk factors known to be associated with T2DM. By including only variables available in the ERICA dataset, we ensured the feasibility and applicability of the predictive model within this specific population.

**8. Provide more detail on validation processes and acknowledge the limitations of the chosen model, including confounding factors**

To address this, we modified the Model Validation topic to provide more detail on the validation process between lines 252-257: *“Internal validation was conducted to identify errors related to data entry and model programming. Biostatistics experts peer reviewed the methods and the scripts, ensuring methodological rigor. Experts with knowledge in epidemiology and on ERICA Study confirmed the appropriateness of applying the model to its sample. In the absence of follow-up data on ERICA’s sample clinical outcomes, it is not possible to assess whether individuals progressed to T2DM as predicted.”*

Also, we have added subsection 2.5 Limitations to the Methods section between lines 258 and 274 to acknowledge potential confounding factors and model limitations:

“*There are limitations to the application of the selected model to ERICA’s sample. First, the composition of both samples is similar, but their baseline age differs, which can impact on their clinical evolution. In addition, while the selected model was based in a study that followed cardiovascular risk factors also observed on ERICA Study, the location in which they were both conducted where different, and they started over 30 years apart. As the development of T2M relates to a person’s habits, such discrepancies limit the comparability of the samples.*

*Certain potential confounding factors — such as socioeconomic status, dietary habits, genetic predisposition, and access to healthcare — were not incorporated into the model. While these factors are known to influence T2DM risk, they were not tracked or incorporated into the original model, so their exclusion was necessary to maintain data reliability and comparability with the reference study. This approach minimizes subjectivity and ensures that the projections are based solely on CVRF that have been consistently measured. Moreover, the model was developed to consider the full period of primary study, so it is limited to its duration and does not permit intermediate evaluations.”*

**9. In the result section, its better to explain more about sensitivity analyses. For example, the results indicate a wide range (1.1% to 28%) in the sensitivity analyses. The implications of this broad range for public health policy and intervention strategies could be discussed more thoroughly.**

We have expanded Discussion section to provide more details on how the significance of the sensitivity analysis findings. The revised text, now included in lines 466-473, states: *“Probabilistic sensitivity analysis indicates the prevalence estimation could range from 1.1% to 28%, depending on the weight applied to cardiovascular risk factors included in the model. However, as cardiovascular disease and diabetes are intertwined, and risk factors are common to both [1, 2, 4], it is unlikely for all CVRF to have the lower limit weight on the development of T2DM. Having some sort of interval is still useful given the limitations of the analysis, the natural heterogeneity of the population, and the multifactorial influences on the development of the disease, as they inform decision-makers on the possible different scenarios.”* This revision ensures that the broad range in sensitivity analyses is contextualized within public health relevance, highlighting that while variability is expected, it does not negate the predictive value of the model. Instead, it underscores the complexity of T2DM risk estimation and the importance of considering multiple influencing factors when interpreting projections for policy and intervention planning.

**Reviewer #3**

**Introduction (lines 117-118): The study aim is to analyse the prevalence of T2DM in the ERICA population or use ERICA´s data to predict the future prevalence of diabetes? Please review the aim of the study to make it clear for the readers.**

We appreciate the reviewer’s comments regarding the clarity of the study’s aim. The study’s objective is not to analyze the current prevalence of T2DM in the ERICA population, addressed in other studies, but rather to use ERICA’s data to predict its future prevalence based on cardiovascular risk factors identified during adolescence. To ensure this distinction is clear, we have revised the text to explicitly reflect the predictive nature of the study.

The abstract was slightly modified, so that the sentence on the study’s goal was changed from lines 34 to 36: *“This paper aims to estimate the future prevalence type 2 diabetes mellitus on individuals from the Study of Cardiovascular Risk Factors in Adolescents (ERICA) sample, based on the progression of their cardiovascular risk factors”*

The revised introduction now reads on lines 127-129: “*Given the increasing impact of T2DM on public health, this study aims to estimate its future prevalence on ERICA’s representative sample of adolescents by projecting the progression of their baseline CVRF and the resulting likelihood of developing T2DM.*”

**Table 7: I suggested changing the name of the column “Individuals with T2DM by state” to “Estimated number of individuals with T2DM” to avoid confusion. The current name of the column make it think that this is the actual number of individuals with diabetes in the ERICA study data. Additionally, it is not necessary to say that is by state because the table is already showing the values by each state.**

We have revised "Individuals with T2DM by state" to "Estimated number of individuals with T2DM", ensuring it accurately reflects that these values are projections from the predictive model rather than observed cases from the ERICA dataset. Additionally, as the table already lists data by state, we have removed the redundant "by state" phrasing in the column headers. These changes improve the precision of the table’s presentation and prevent potential misinterpretation.

**Results (Line 340): It is not clear how the period of 20.5 years were considered in the model. Is this a parameter in the model?**

This information was already addressed in the manuscript, as the model was based on the Bogalusa Heart Study, which inherently follows individuals over this timeframe. Nonetheless, we recognize that this connection may not have been immediately clear to all readers. To reinforce this point, we have revised the Discussion section to explicitly state that the model's projections are inherently constrained by the Bogalusa study’s follow-up period. The revised text on this topic now reads on lines 485-490: *“Furthermore, the model was developed with data from Bogalusa’s cohort, which was monitored for an average of 20.5 years. As a consequence, it is challenging to extrapolate these findings to larger timeframes. Given that age is a known risk factor for T2DM and considering the mean age of ERICA participants at baseline, the projections may underestimate the disease incidence within this sample”.*

**Discussion: The authors considered only biological factors in the predictive model of T2DM, but it is already known that social factors are also important in the development and progression of this disease. The authors could also include this topic on the discussion when describing the importance of having a predictive model developed to the Brazilian population. This model could then consider the socioeconomic factors (i.e. income, education, housing) to better predict the future occurrence of type 2 diabetes.**

Our model focused exclusively on clinical variables, we acknowledge that socioeconomic factors such as income, education, housing, access to food, and healthcare play a significant role in shaping long-term health outcomes, including diabetes risk. To address this, we have incorporated a discussion on the potential influence of social determinants and the limitations of not including them in our predictive model. The revised text now states on lines 493-501: " *It is also not possible to understand if there have been any interventions in Brazil during the growth of ERICA’s individuals that could have changed their probability of developing T2DM. Also, the model exclusively included clinical variables, while there is extensive literature on how social determinants of health influence the progression of T2DM, such as income, education, housing, access to food and healthcare. Differences in the Bogalusa and ERICA samples regarding these determinants are not quantifiable but might also limit the interpretation of results. Future studies could incorporated socio-economic factors in addition to the clinical ones, to enable a better understanding of the Brazilian reality [22].*" This revision ensures that we acknowledge the broader context of diabetes development and recognize the limitations of our approach, while also suggesting future research directions that could refine predictive models for the Brazilian population. In addition, it includes a sentence to acknowledge that the model does not incorporate eventual policy interventions in Brazil.
